# The Probabilities of Unique Events

**DOI:** 10.1371/journal.pone.0045975

**Published:** 2012-10-02

**Authors:** Sangeet S. Khemlani, Max Lotstein, Phil Johnson-Laird

**Affiliations:** 1 Navy Center for Applied Research in Artificial Intelligence, Naval Research Lab, Washington, District of Columbia, United States of America; 2 Department of Psychology, Princeton University, Princeton, New Jersey, United States of America; University of Leicester, United Kingdom

## Abstract

Many theorists argue that the probabilities of unique events, even real possibilities such as President Obama's re-election, are meaningless. As a consequence, psychologists have seldom investigated them. We propose a new theory (implemented in a computer program) in which such estimates depend on an intuitive non-numerical system capable only of simple procedures, and a deliberative system that maps intuitions into numbers. The theory predicts that estimates of the probabilities of conjunctions should often tend to split the difference between the probabilities of the two conjuncts. We report two experiments showing that individuals commit such violations of the probability calculus, and corroborating other predictions of the theory, e.g., individuals err in the same way even when they make non-numerical verbal estimates, such as that an event is *highly improbable*.

## Introduction

Everyone from Aristotle to aboriginals engages in probabilistic thinking, even if they know nothing of the probability calculus. In April 2012, we judged the probability that this paper would appear in *PLOS ONE* to be 0.1. For *frequentists* and evolutionary psychologists, who interpret probabilities as the limits on the frequencies of repeated observations, such a probability is meaningless [1]–[3]. It is has no obvious truth conditions, i.e., circumstances in which it would be true and circumstances in which it would be false. But, for *Bayesians*, who interpret probabilities as degrees of subjective belief, our estimate is meaningful because individuals have beliefs about unique events and should naturally assign probabilities to them [4]–[7]. Various methods exist to test whether these estimates truly reflect an individual's beliefs [4], [6]. In previous studies, notably those of Tversky and Kahneman [8] participants estimated the probabilities of unique events concerning imaginary scenarios, such as:

Linda is 31 years old, single, outspoken, and very bright. She majored in philosophy. As a student she was deeply concerned with issues of discrimination and social justice and also participated in antinuclear demonstrations.

The participants ranked the probability that Linda is a feminist bank teller as higher than the probability that Linda is a bank teller. The description is more representative of the former than the latter. Frequentists retorted that such a flagrant violation of the probability calculus was a result of a psychological experiment that obscured the rationality of the participants, and that the norms of the calculus are relevant only to judgments about naturally occurring frequencies [1], [9].

We show that naive individuals violate the probability calculus in simple estimates of real possibilities, not just in scenarios contrived to elicit the use of the representativeness of a description as a guide to its probability. Previous studies have seldom examined estimates of such probabilities, e.g.:

What is the chance that Obama is reelected President in November?

As our theory predicts, they too lead to systematic errors. A major mystery about such estimates is the mental operations that underlie them, and an even bigger mystery is where the numbers come from and what determines their magnitudes. To solve these mysteries, we developed a theory based on mental models 10,11 and, unlike previous accounts of the psychology of probabilities, we have implemented the theory in a computer program that yields estimates of the probabilities of unique events. The theory and its computer implementation predict the occurrence of violations of the probability calculus both in numerical and in verbal estimates of probabilities. We report two experiments corroborating the theory's predictions, and so we begin with a description of the theory.

### A theory and computational model of subjective probabilistic reasoning

Suppose that you are asked the question about the possible re-election of Obama; what estimate would you give? At the time of writing, you are likely to estimate a probability of around 54% (as evinced in on-line betting sites, such as intrade.com). A numerical estimate comes to mind quite readily for most individuals, but the process – according to the present theory – is quite complex, and depends on two separate components, an intuitive pre-numerical component and a deliberative component that carries out arithmetic and that maps intuitions into numerical estimates. This sort of distinction is familiar in “dual process” accounts of reasoning and decision making [12]–[16], which hark back to still earlier antecedents in Turing [17] and Pascal [18]. What distinguishes the present account, however, is that the distinction is drawn in terms of computational power, and that both the intuitive and deliberative systems have been implemented as part of a computational model of reasoning, mReasoner v. 0.9, which unifies deductive and probabilistic reasoning. Its source code is available at: http://mentalmodels.princeton.edu/models/mreasoner/. In what follows, we describe the theory and illustrate its workings with examples from the computer program.

The intuitive system constructs an iconic, non-numerical magnitude representing the strength of belief in a proposition. The first step in estimating, say, the probability of Obama's re-election is to call to mind relevant evidence, such as:

Most incumbent US Presidents are re-elected.

According to the theory, mental models represent possibilities [19], [20], and so the theory postulates that individuals build a single mental model of a set of individuals to represent this belief:
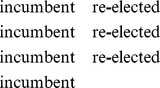



Each row in this diagram represents an incumbent, and so the first row represents an incumbent who is re-elected, as do the second and third rows, and the last row represents an incumbent who is not re-elected. Mental models, of course, represent individuals, and we use words in the diagram above solely for convenience. The absolute numbers of individuals in the model are not fixed, and during inference they can be modified, or even tagged with numerical values, provided that the result does not contravene the meaning of the quantified assertion as embodied in a separate “intensional” representation [21]. The proportion of incumbents in the model who are re-elected represents the quantifier, *most of the incumbents*. Models of quantified assertions of this sort are independently supported from evidence on how individuals reason from them [22]. Because Obama is an incumbent US President, the model can be sampled to yield an iconic representation of the probability of Obama's re-election.

The intuitive pre-numerical system yields an analog magnitude monotonically related to the proportion of possibilities in the mental model in which Obama is re-elected. We refer to this system as “pre-numerical” because it uses a representation of numbers of the sort that is found in infants [23], [24], animals [25], and adults in non-numerate cultures [26], but that continues to exist in adults in Western cultures too [27], [28]. The computer implementation of the theory uses an internal representation that corresponds to a simple line within two boundaries: 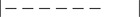



The left vertical represents impossibility, the right vertical represents certainty, and the proportional length of the line represents the probability of the event. This representation can be translated into a verbal estimate, such as:

The re-election of Obama as US President is *highly likely*.

The theory assumes that individuals can draw from more than one source of evidence and accordingly alter their degree of belief. Some evidence may already be in the form of a probability. But, other evidence may not be, e.g.:

Few Presidents during economic recessions are re-elected.

The mental models of this evidence can also yield an analog representation:
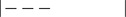



In isolation this representation yields an evaluation of the re-election as *improbable*. But, in the case that individuals adduce both pieces of evidence, how should they combine them rationally? Those unfamiliar with the probability calculus do not know the answer to this question.

In general, given P(*A*|*B*) and P(*A*|*C*), the problem is to determine P(*A*|*B&C*). For instance, if P(*A*|*B*) is 75% and P(*A*|*C*) is 33%, then what is the probability of P(*A*|*B&C*)? If no other information is available, then it is impossible to give a rational answer, because the probability can vary from 0 to 1. A rational estimate depends on an extension of Bayes's theorem and therefore on gathering evidence about other probabilities. But, naive individuals do not have this knowledge. So, how do they combine such estimates?

The answer to this question reflects a fundamental aspect of the pre-numerical system. It has no access to working memory, and so it can hold at most one icon representing a belief [13], and is capable only of simple pre-numerical procedures on iconic representations. Hence, without access to working memory, which allows for a potentially infinite number of states, the pre-numerical system has no more computational power than a finite-state automaton [29]. It is embodied in mReasoner and can repeat operations, but only a small finite number of times, and this constraint severely curtails the sorts of process that it can carry out on icons representing beliefs, e.g., it can multiply them only in a primitive way. When two sources of evidence conflict, as in the case of Obama's re-election, the pre-numerical system has a limited number of options in resolving them. Individuals unfamiliar with the probability calculus should tend to treat the conflict as calling for a compromise. The program integrates the representations of the two degrees of belief by establishing a pointer, ?, to the second belief within the representation of the first:
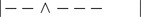



A simple computation of a compromise is to shift the pointer towards the right-hand end of the line and at the same time to shift the end of this line towards the pointer until they meet, i.e., the result of splitting the distance roughly halves it. And the point where they meet is the new degree of belief that takes into account both pieces of evidence: 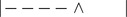



An analogous problem occurs when individuals have to estimate the probability of a conjunction, P(*A&B*), such as:

What is the likelihood that US unemployment declines by several percentage points this year and that Obama is re-elected President?

Once again, the intuitive system has only a limited number of options in coping with conjunctions. One option is to treat them in the same way as separate pieces of evidence, that is, as calling for a compromise. Hence, an icon representing the probability of the preceding conjunction, in effect, splits the difference between the intuitive probabilities of its respective conjuncts. Some of our unpublished experiments suggest that this procedure is used for other sorts of compound assertions too, even inclusive disjunctions. Naive individuals confuse uncertainty with improbability: a disjunction creates uncertainty, and so in error they take it to be more improbable than one or both of its disjuncts.

Another option is available to the pre-numerical system, and embodied in mReasoner. It can be illustrated in the case of a *highly improbable* estimate for a decline in US unemployment but a *probable* estimate for Obama's re-election. Individuals may be able to make an intuitive allowance for the relative improbability of the conjunction of both events. The intuitive system accordingly allows a primitive finite form of multiplication in which the length of an icon representing one belief is used to take a proportion of another. Again, the system for making this estimate is limited to a small number of repeated operations.

In contrast to the intuitive system, the arithmetical system makes use of a working memory for the results of intermediate computations [29], and it maps analog magnitude representations of beliefs, such as:
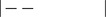



into numerical values, such as: 25%. Its conversions are subject to error, which is an inevitable consequence of mapping icons into a fine numerical scale. A corollary is that the mapping can err without yielding a conjunction fallacy, e.g., P(*A*) = 70%, P(*B*) = 75%, and P(*A&B*) = 40%, which yields P(*¬A*&*¬B*) = −5%. In other words, given estimates of P(*A*) and P(*B*), an estimate of their conjunction can yield a conjunction fallacy, or, even without such a fallacy, it can yield a negative probability as in the preceding case for P(*¬A*&*¬B*). Dirac introduced negative probabilities into quantum mechanics, and some psychologists have argued that they have a role to play in accounting for errors in judgment [30]. But, in estimates of everyday events, they are straightforward errors: nothing is less probable than the impossible.

Once individuals map iconic representations to numerical ones, they can in principle hold the numerical estimates in working memory and make more sophisticated estimates of the probability of conjunctions. If you estimate the chance of Obama's re-election as 60%, and the chance of an economic recovery in the USA as 40%, then you might take 60% of 40%, or vice versa, as your estimate of their joint occurrence. Such multiplicative estimates are in accordance with the probability calculus provided that the two events are independent – a condition that the Obama example violates, and so it calls for the computation of P(*A*) * P(*B|A*).

The arithmetical system can try to keep track of the complete *joint probability distribution* (henceforth, the JPD), i.e., the respective probabilities of each of the exhaustive set of conjunctions between events. For instance, for two events, *A* and *B*, the conjunctions in the JPD are P(*A&B*), P(*A&¬B*), P(*¬A&B*), P(*¬A&¬B*), where “*¬A*” denotes that *A* does not occur. The mapping from intuitive icons to a scale is more likely to yield a consistent JPD with a coarse scale than with a fine scale. A slight jitter in mappings to a coarse scale should not tend to change categories from a consistent to an inconsistent JPD, whereas such a change is more likely with a fine scale. This argument is corroborated in a Monte Carlo simulation. A random assignment of values on a seven-point scale for P(*A*), P(*B*), and P(*A&B*) yields a consistent JPD on about 40% of occasions, whereas a random assignment of values on a scale ranging from 0 to 100 yields a consistent JPD on only 33% of occasions. Hence, a coarse verbal scale for probabilities is biased to yield a greater proportion of consistent JPDs than a fine numerical scale.

In sum, the theory makes four main predictions about the estimates of the probabilities of real but unique possibilities:

1. Participants from the same population have access to roughly the same sorts of evidence about real possibilities, and so their estimates should concur reliably.
2. Estimates of the conjunctions of events should yield frequent violations of the JPD, where a violation is defined as a negative probability in at least one of the four probabilities that comprise the JPD, i.e., P(*A&B*), P(*A&¬B*), P(*¬A&B*), P(*¬A&¬B*). In the studies described below, reasoners estimated P(*A*), P(*B*), and P(*A&B*), and this triplet of estimates can be used to fix the probabilities that comprise the JPD. Other triplets can also fix the JPD.
3. Violations of the JPD should be reduced if individuals have already made numerical estimates of the probabilities of the respective conjuncts, because these estimates allow them to use more sophisticated numerical estimates, such as taking a percentage of a percentage, i.e., a “multiplicative” estimate. Such estimates, of course, are irrational in the case that the two conjuncts are not independent.
4. Violations of the JPD should also occur, but to a reduced degree, with a verbal scale of probabilities in comparison with a full percentage scale.

We carried out several experiments to test these predictions, and report the two most important and representative of them, but their principal results have been replicated in other studies.

## Methods

### Experiment 1: Conjunctive probability estimates

Experiment 1 tested three predictions: first, participants should concur in their estimates of the probabilities of unique events; second, their estimates should frequently violate the JPD; and, third, these errors should be reduced in favor of more sophisticated multiplicative procedure when participants have already made numerical estimates of the likelihoods of the conjuncts before they estimate the likelihood of their conjunction.

#### Participants

39 participants completed Experiment 1 for monetary compensation (a $10 lottery) on Amazon Mechanical Turk, an online platform hosted on Amazon.com [31]. All of the participants stated that they were native English speakers.

#### Design and procedure

Table 1 shows the contents of a typical trial. For each problem, the participants provided three probability estimates concerning two unique events: P(*A*), P(*B*), and their conjunction, P(*A&B*). On half of the trials, participants estimated the probabilities in the order P(*A*&*B*), P(*A*), and then P(*B*); and on the other half of the trials, they estimated the probabilities in the order P(*A*), P(*B*), and then P(*A*&*B*). They carried out 16 problems in total. Table 2 shows the contents of the problems, which were drawn from five different domains and concerned real unique possibilities in sports, science, economics, politics, and entertainment. In order to examine the possibility of systematic effects of contents, half the contents of the problems were such that in the conjunction *A and B*, *A* was likely to increase the probability of *B*, and in the other half, *A* was likely to decrease the probability of *B*. This difference was established in a prior norming study of 34 sentences. Hence, *A* and *B* were not independent events. The order of the problems and the assignment of contents were randomized, and participants encountered a particular content only once in the experiment. The participants were instructed that they were to make a sequence of sets of estimates of the likelihoods of events, and that they could take as much time as they needed. On each trial, the participants estimated sets of three probabilities. The program administering the experiment presented each question separately, and it recorded the participant's numerical estimate and its latency.

**Table 1:** An example of a problem in Experiment 1, and its abstract form.

Order	Question	Probability estimate
1	What is the probability that a nuclear weapon will be used in a terrorist attack in the next decade?	P(*A*)
2	What is the probability that there will be a substantial decrease in terrorist activity in the next 10 years?	P(*B*)
3	What is the probability that a nuclear weapon will be used in a terrorist attack in the next decade *and* there will be a substantial decrease in terrorist activity in the next 10 years?	P(*A&B*)

Participants responded to questions 1–3 with numerical estimates ranging from 0 through 100.

*Note: A* = nuclear attack, *B* = decrease in terrorism.

**Table 2:** The conjunctive events of the 16 contents for the problems in Experiment 1, and their respective mean percentage probability estimates.

Conjunctive events (preceded by “What is the probability that…”)	P(*A*)	P(*B*)	P(*A&B*)
*Event A decreases likelihood of Event B*			
…the United States will sign the Kyoto Protocol and commit to reducing CO2 emissions and global temperatures reach a theoretical point of no return in the next 100 years?	47	42	44
…US companies focus their advertising on the Web next year and the *New York Times* becomes more profitable?	69	41	42
…intellectual property law in the US will be updated to a reflect advances in technology by the year 2040 and Russia will become the world center for software development by 2040?	54	24	27
…a nuclear weapon will be used in a terrorist attack in the next decade and there will be a substantial decrease in terrorist activity in the next 10 years?	39	27	26
…the United States adopts an open border policy of universal acceptance and English is legally declared the official language of the United States?	15	46	26
…Greece will make a full economic recovery in the next 10 years and Greece will be forced to leave the EU?	33	33	25
…scientists will discover a cure for Parkinson's disease in 10 years and the number of patients who suffer from Parkinson's disease will triple by 2050?	39	32	25
…Honda will go bankrupt in 2012 and Ford will go bankrupt before the end of 2013?	19	23	15
*Event A increases likelihood of Event B*			
…a new illegal but synthetic drug becomes popular in the USA over the next two years and the movement to decriminalize drugs doubles its numbers by 2015?	58	48	49
…3-dimensional graphics will be required to contain explicit markers to indicate their unreal nature by 2020 and competitive video game playing will achieve mainstream acceptance by 2020?	41	52	45
…the Supreme Court rules on the constitutionality of gay marriage in the next 5 years and a gay person will be elected as president in the next 50 years?	65	40	38
…a significant upturn in the economy occurs next year and Obama will be reelected President in 2012?	36	55	38
…in less than 15 years, millions of people will live past 100 and advances in genetics will end the shortage of replacement organs in the next 15 years?	36	38	37
…space tourism will achieve widespread popularity in the next 50 years and advances in material science will lead to the development of anti-gravity materials in the next 50 years?	34	40	36
…at least one head of state will be assassinated by 2012 and NATO will grant military support to Arab Spring movements in several countries?	39	36	32
…intelligent alien life is found outside the solar system in the next 10 years and world governments dedicate more resources to contacting extra-terrestrials?	20	18	17

The table presents the contents in which *A* decreased the likelihood of *B*, and then the contents in which *A* increased the likelihood of *B*.

### Experiment 2: Verbal and numerical probability estimates

Experiment 2 used the same method as the preceding experiment to test the theory's prediction that an inconsistent JPD should occur in both verbal and numerical estimates, but tend to be greater with numerical estimates because of the use of a finer scale. The verbal judgments were on a 7-point ordinal scale: *Impossible, Highly improbable, Improbable, As likely as not, Probable, Highly probable, Certain*. The numerical estimates, as in the previous experiment, were of the chances out of 100, ranging from 0 through 100 in integer steps. Participants made four probability estimates for each problem, and there were four forms of problem designed to mask the relation between the conjunctions and their conjuncts.

#### Participants

18 participants completed Experiment 2 for monetary compensation on the same online platform as in the previous study. All of the participants stated that they were native English speakers.

#### Design and procedure

To mask the relation between the conjunctions and their conjuncts, participants made four probability estimates for each problem, and there were four forms of problem:

P(A&B), P(A), P(B)

P(¬A&B), P(¬A), P(B)

P(A&¬B), P(A), P(¬B)

P(¬A&¬B), P(¬A), P(¬B)

In each case, there was a fourth judgment corresponding to the probability of a conjunction of the respective negations of the two propositions in the initial conjunction. As in the previous study, half the problems were those in which *A* increased the probability of *B*, and half the problems were those in which *A* decreased the probability of *B*. The participants carried out the estimates in two blocks of 8 problems, one verbal and one numerical, and the order of the two blocks was counterbalanced. Hence, there were 16 sorts of trial as a function of the task (verbal or numerical), the four forms of problem, and the two sorts of relation between *A* and the probability of *B*. Each of the 18 participants carried out one trial of each sort with different contents, which were presented in a random order. The procedure and the contents of the problems were identical to those of Experiment 1 with the exception that each trial called for four sorts of judgment.

## Results

### Experiment 1: Conjunctive probability estimates

As Table 2 suggests, the participants concurred in the rank order of the probabilities that they estimated for the P(*A*) events, for the P(*B*) events, and for their conjunction P(*A&B*) (Kendall's W's = .33, .18, .20, respectively, p<.0001 in all three cases). This result corroborates the theory's first prediction: participants have access to evidence in common, which they adduce in making their estimates. Overall, their estimates of P(*A&B*), P(*A*), and P(*B*) violated the JPD on 56% of the trials, and every one of the 39 participants made one or more such estimates (Binomial test, p<.0001). Indeed, 27 out of 39 participants violated the JPD on 50% of trials or more (Binomial test, p<.025). These results corroborate the second prediction: violations of the JPD are frequent. Table 3 presents the percentages of participants' violations of the JPD, and the latencies of their estimates, depending on the two orders of the estimates. The participants were more likely to violate the JPD when the conjunction occurred first (62% of violations) than when it occurred last (51%; Wilcoxon test, z = 2.16, p<.025). This result corroborates the third prediction. The pattern of latencies in Table 3 shows that participants took longer to estimate the probability of a conjunction when it occurred first than when it occurred last (12.79 s vs. 7.55 s, Wilcoxon test, z = 4.76, p<.0001). When the conjunction occurs first, they have to think about each event, and their conjunction; when the conjunction occurs last, they have already thought about each event and estimated their likelihoods, and so they need to think solely about their conjunction.

**Table 3:** The two different orders of estimates in Experiment 1, the percentage of participants' violations of the JPD, and the latencies (in s) of participants' estimates of the three different probabilities.

		Latency of estimates (in s)		
Order of estimates	Percentage of violations of JPD	P(*A*)	P(*B*)	P(*A&B*)
P(A), P(B), P(A&B)	51	8.46	7.53	7.49
P(A&B), P(A), P(B)	62	6.47	5.84	12.77

**Figure 1:**
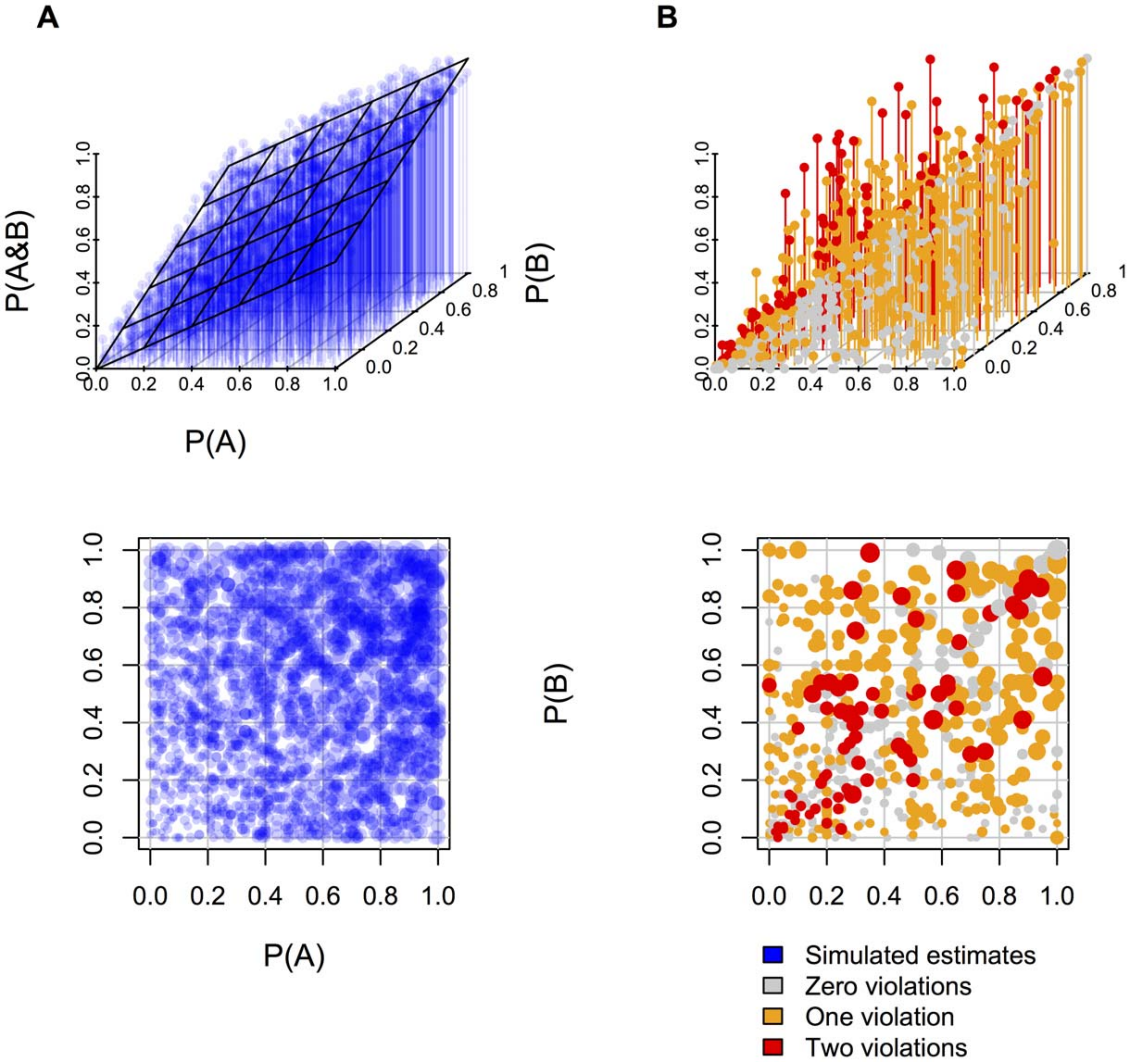
3D scatterplots of estimates of P(*A*), P(*B*), and P(*A&B*) and 2D scatterplots of estimates of P(*A*) and P(*B*) in Experiment 1. Panel A shows the estimates of 2000 simulated runs of the computational model and its best fitting linear regression plane, and Panel B shows participants' estimates. Participants' estimates were separated by whether the estimate reflected zero, one, or two violations of the JPD. A violation was defined as a negative probability in the JPD extrapolated from the estimates. In the 2D scatterplots, estimates of P(*A&B*) correspond to the size of points such that larger points indicate larger estimates.

Figure? 1 presents scatterplots of the relations between the estimates of P(*A*), P(*B*), and P(*A&B*). Figure 1a shows the predictions of the computational model for the conjunctive probability given the participants' estimates of the two conjuncts. The computational model (Figure 1a) yields a close fit to the data from Experiment 1 (Figure 1b), and shows that individuals often tended to evaluate P(*A&B*) by splitting the difference between P(*A*) and P(*B*). The R^2^ value for the fit between the theory and the data was .76, p<.0001, and the root mean squared error (RMSE) was .14. When participants did not violate the JPD, they tended to multiply the two estimates of the conjuncts, and they did so on 28% of trials (and 27 out of 39 participants did so on at least 10% of the trials). We classified a set of estimates as using a multiplication strategy if two constraints were met. First, P(*A&B*) had to be less than or equal to P(*A*) and less than or equal to P(*B*). Second, the difference between the P(*A&B*) estimate had to be within 5% of the computed multiplicative estimate, P(*A*) * P(*B*). The multiplicative response would have been correct if the two events were independent of one another, but they were not.

The study revealed an effect of content. If event *A* decreases the probability of event *B*, then individuals should tend to make a lower estimate for the probability of both events than if event *A* increases the probability of event *B*. A lower probability for the conjunction, in turn, is less likely to yield a violation of the JPD. The results corroborated this effect: a decrease in the probability of *B* yielded 52% violations, whereas an increase in its probability yielded 61% of violations (Wilcoxon test, z = 2.14, p<.025). After the participants had estimated the probability a conjunction, P(*A&B*), they might have supposed that the task of estimating the probability of a conjunct, P(*A*), called for them to estimate P(*A&¬B*). Even on this assumption, however, the participants violated the JPD on 49% of the problems (Wilcoxon test, z = 5.53, p<.0001). Moreover, the re-interpretation of the estimate of a conjunct, P(*A*), is very unlikely when the estimate occurs first in the problem, because the participants have yet to encounter the proposition, *B*. The participants varied enormously in their tendency to make estimates that violated the JPD: the best participant in the experiment made no such estimates, whereas the worst participate made only such estimates. We suspect that the cause of such a vast divergence is the well-known difference from one individual to another in relying on intuition [14], [15] and perhaps in their familiarity with the probability calculus.

### Experiment 2: Verbal and numerical probability estimates

**Figure 2:**
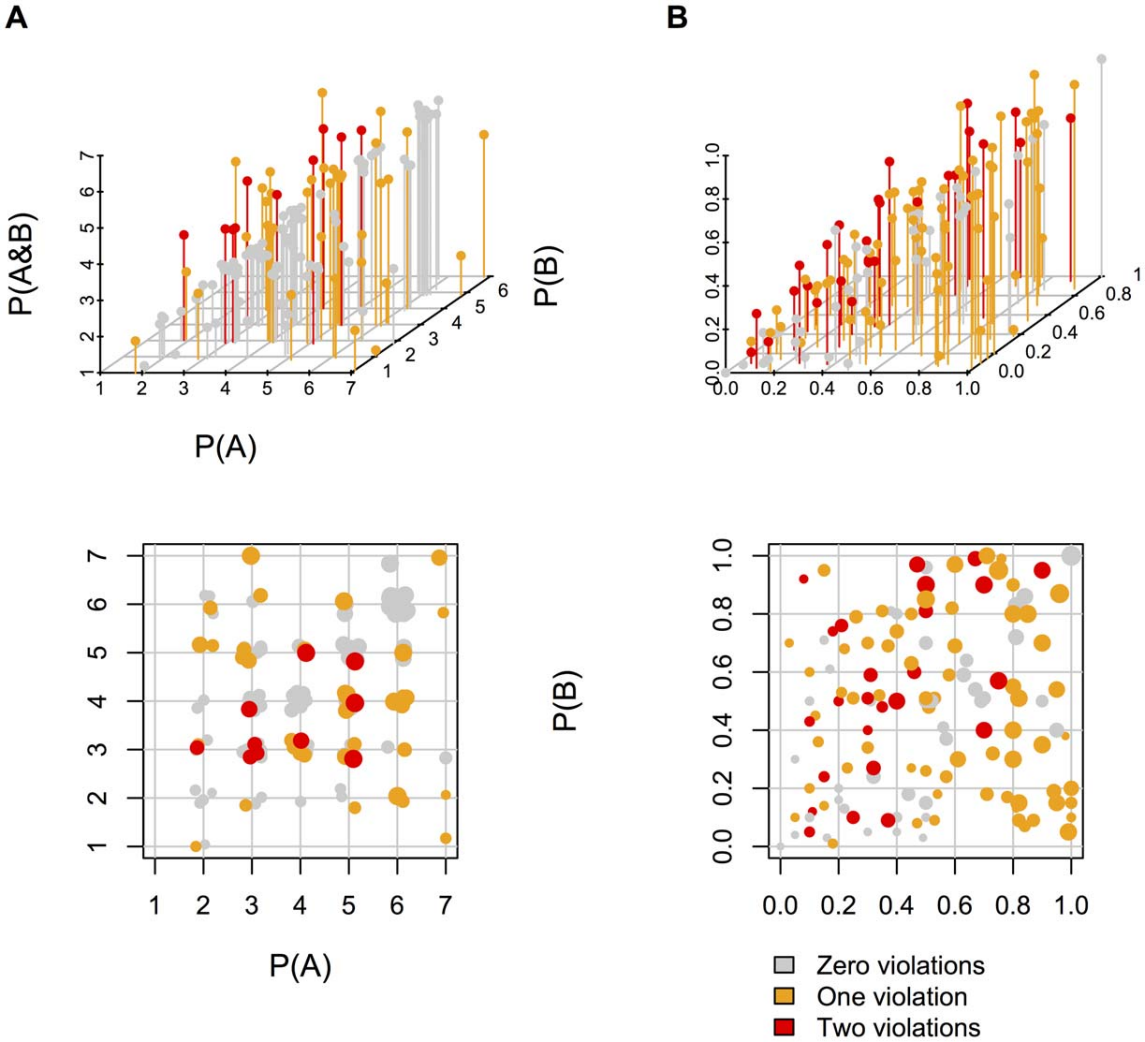
3D scatterplots of estimates of P(*A*), P(*B*), and P(*A&B*) and 2D scatterplots of verbal scale (Panel A) and numerical scale (Panel B) estimates of P(*A*) and P(*B*) in Experiment 2 (see **Figure 1** for an explanation of zero, one, or two violations). Participants' estimates were separated by whether the estimate reflected zero, one, or two violations of the JPD. A violation was defined as a negative probability in the JPD extrapolated from the estimates. In the 2D scatterplots, estimates of P(*A&B*) correspond to the size of points such that larger points indicate larger estimates.

Participants violated the JPD on 34% of their verbal judgments and on 68% of their numerical judgments (Wilcoxon test, z = 3.55, p<.0005) even though their verbal estimates were faster than their numerical estimates (65.9 s to estimate all four probabilities vs. 85.1 s, Wilcoxon test, z = 3.33, p<.001). These results corroborated the prediction that violations should occur in verbal estimates, but at a reduced rate because of the relative coarseness of the scale. The four different sorts of problem yielded no reliable differences in participants' tendency to yield inconsistent JPDs (Friedman test, χ^2^ = 4.19, p = .12), and the two different relations between *A* and the probability of *B* did not reliably affect the tendency, either (Wilcoxon test, z = .16, p = .88). Figures 2a and 2b show scatterplots of the verbal and numerical estimates. They too show that individuals have a predictable tendency to split the difference between the two probabilities in order to estimate the likelihood of their conjunction (R^2^ = .31, p<.0001, and .67, p<.0001, and RMSE = .26 and .15, for the verbal and numerical estimates, respectively).

## Discussion

The mechanisms underlying naive estimates of the probabilities of unique events are largely inaccessible to consciousness, but they are open to psychological investigation. We proposed a model-based theory, which was designed to solve the mystery of the mental operations underlying these estimates, and the deeper mystery of where the numbers come from and what determines their magnitudes. Like other theories of judgment and reasoning [12]–[16], this theory distinguishes between an intuitive pre-numerical system and a deliberative system capable of arithmetic. But, unlike other accounts, the present theory distinguishes the two systems both in computational power – only the deliberative system has access to working memory – and in implementing them as part of a computer program. This program, mReasoner, provides a unified account of deduction and probabilistic reasoning. The intuitive system uses mental models of evidence to construct iconic representations of degrees of belief. It can carry out only pre-numerical operations on these icons, such as splitting the difference between them, or at best using one degree of belief as a rough proportion of another – a primitive form of multiplication. The iconic representations support only intuitive verbal descriptions of beliefs, such as: President Obama is *likely* to be re-elected. The deliberative system has access to working memory, and so it can map icons into numerical estimates, and it can multiply probabilities exactly.

Two experiments corroborated the main predictions of this theory and its implementation. They showed that individuals tend to concur in the rank order of their estimates of the probabilities of unique events (prediction 1). For example, they inferred that the US is much less likely to adopt an open border policy (mean estimate: 15%) than to make English the official language of the country (mean estimate 46%; see Table 2). The participants therefore share at least some common knowledge and systematic principles to make these estimates, and so, contrary to frequentists [1]–[3], the probabilities of unique events are not meaningless. Participants often estimated the probability of a conjunction of two unique events by splitting the difference between their estimates of the probabilities of the two events (prediction 2). For example, their mean estimate of the conjunction of the US adopting an open border policy and making English the official language was 26%, a value falling between their mean estimates of the two conjuncts (see Table 2). Experiment 1 varied the order of the estimates, and violations of the JPD were reliable smaller when the conjunction came last as opposed to first (prediction 3). When the conjunction was last, the participants had already made numerical estimates of the probabilities of its conjuncts, and so they could use a deliberative procedure, such as taking a percentage of a percentage, i.e., a “multiplicative” estimate. Such estimates, of course, are unwarranted in the case of the experimental materials because, as a prior study showed, the two conjuncts were not independent. Experiment 2 compared purely verbal estimates with numerical estimates, and it showed that systematic violations still occurred, but to a reduced degree, with a seven-point verbal scale of probabilities in comparison with a full percentage scale (prediction 4).

Could splitting the difference be an artifact, or a result of the participants merely guessing probabilities? Two results suggest otherwise. First, the reliable concordances of the estimated probabilities showed that the participants were relying to some degree on beliefs and procedures in common. Second, the large and reliable increase in time to estimate the probability of conjunctions when these estimates occurred before the estimates of their respective conjuncts (in Experiment 1) showed that the participants were thinking in order to make their estimates, and thought nearly twice as long to estimate P(*A&B*) than to estimate either P(*A*) or P(*B*).

At present, no rival theories propose mechanisms for the estimates of the probabilities of unique but real possibilities. Critics might argue, however, that the role of mental models and belief icons in yielding the present predictions is superfluous. Any theory, whether it represented probabilities with vague verbal quantifiers, or precise numerical values, could simulate the principles of the present theory and succeed as well in accounting for the results. We have two reactions to this claim. On the one hand, of course a theory might be formulated ex post facto to account for our results, but the strength of the model theory is that its principles emerge naturally from its unification of deductive inference and the representation of quantified assertions, such as: *most incumbents are re-elected*, and from its postulation of an intuitive system that lacks the computational power to cope with anything but pre-numerical representations and processes lacking full arithmetic. Splitting the difference is one such option for accommodating both divergent evidence and conjunctions of divergent probabilities. The principles embodied in the theory and its computer implementation yield the four predictions that our experiments corroborated. On the other hand, we can and have compared the model theory with some other extant theories [32]–[34]. These theories were not framed for the probabilities of unique but real possibilities, but to account for results in experiments using assertions about hypothetical cases, such as: *persons rarely possess gene x* [32], estimates of various attributes of a hypothetical individual described in a brief scenario [33] in the tradition of Tversky and Kahneman's studies [8], and estimates of class-membership, such as, the likelihood that a given butterfly is a Monarch [34]. Likewise, these studies were not intended to give an account of underlying mental processes or the origins of the numbers in estimates of probability, e.g., one theory posits that in estimating P(*A&B*) individuals tend to compare numerators and neglect denominators [34]. But, given the values of P(*A*) and P(*B*), two of theories [32], [33] provide formulas for predicting the value of P(*A&B*). Table 4 shows that the computational implementation of mReasoner yields a better fit to the data than these formulas. The system accounts for more variance because it is able to explain both of the strategies that participants tended to use, i.e., the split the difference strategy and the multiplicative strategy.

**Table 4:** Model comparisons (R^2^ and RMSE) between mReasoner and two alternative models of probability estimates (Wyer's [32] equation 3a: {P(A)+P(B)/2+[P(A)*P(B)]}, and Fantino et al. [33] [P(A)+P(B)/2] against the data from Experiments 1 and 2.

	Model fits	
	R^2^	RMSE
*Experiment 1*		
**mReasoner**	**.75**	**.14**
Wyer (1976)	.65	.17
Fantino et al. (1997)	.64	.18
*Experiment 2* (numerical)		
**mReasoner**	**.67**	**.15**
Wyer (1976)	.53	.19
Fantino et al. (1997)	.54	.19
*Experiment 2* (verbal)		
**mReasoner**	**.31**	**.25**
Wyer (1976)	.22	.24
Fantino et al. (1997)	.25	.24

*Note: All R^2^ values were significant, ps<.0001.*

One final issue warrants discussion. Our experiments examined pairs of events, which a previous norming study showed were not independent of one another. Hence, it is natural to wonder what would happen in estimates of the conjunction of independent events [32]. One methodological difficulty is that such conjunctions of real possibilities are unusual and seem quite odd, e.g.:

What is the probability that a cure for Parkinson's disease will be found in ten years and that Greece will leave the EU in the next ten years?

The correct estimate in accordance with the probability calculus is to multiply the probabilities of the two conjuncts. If participants could be persuaded that such questions are sensible, then they should be likely to consider each conjunct independently, and as a result to be biased towards a multiplicative response. At present, mReasoner does not model the putative effects of *A* on the probability of *B*. We manipulated the contents in the two experiments so that *A* raised the probability of *B*, or else lowered it. The manipulation had the expected effect in Experiment 1, but not in Experiment 2, perhaps because of the use of negation in most of the problems. Studies of class-membership show large effects of dependence [34], but its effects in judgments of real possibilities need further investigation.

Frequentists argue that the probability calculus is inapplicable to the probabilities of unique events [1]–[3], and so violations of the JPD are not necessarily irrational. But, as Bayesians reply, those who violate the JPD are vulnerable to a so-called “Dutch” book, i.e., a set of related bets in which they are bound to lose money if their bets reflect such violations [4], [6], [35]. Individuals often make bets on estimates of real unique possibilities, including the re-election of Obama, using so-called “prediction markets”, e.g., intrade.com, so it is in their interest to make rational probability estimates. In conclusion, the present research shows that naive individuals readily provide both numerical and non-numerical estimates of the probability of unique events, such as that Greece will be forced to leave the EU in the next ten years, and they show a reliable concordance in their estimates over different events. So, they rely on a tacit and organized system for such estimates, which we have modeled computationally. More importantly, this model predicts a systematic tendency to split the difference in estimating the probability of conjunctions, and, as a result, to estimate a probability for them that violates the JPD. Our studies bore out the prediction. The phenomenon is robust and novel, because violations of the probability calculus have not previously been shown to occur in estimates of the probabilities of real but unique possibilities. It also corroborates our hypothesis that these probabilities derive from a system that uses a primitive non-numerical representation of numbers akin to a system known to exist in infants and other animals.

## Acknowledgments

We are grateful for helpful criticisms from Sam Glucksberg, Adele Goldberg, Hua Gao, Geoffrey Goodwin, Matt Johnson, Olivia Kang, Dan Osherson, and Laura Suttle. We are also grateful to Vittorio Girotto, Michel Gonzalez, and especially Nuria Carriedo, for collaborating with the third author in some preliminary studies of the probabilities of unique events.
